# Effects of melatonin on testicular function in adult male mice under different photoperiods

**DOI:** 10.1590/1984-3143-AR2022-0038

**Published:** 2022-09-26

**Authors:** Dan-li Jiang, Yang-long Xu, Jian-qiu Pan, Di Fan, Xu Shen, Wan-yan Li, Hong-jia Ou-Yang, Dan-ning Xu, Yun-bo Tian, Yun-mao Huang

**Affiliations:** 1 College of Animal Science & Technology, Zhongkai University of Agriculture and Engineering, Guangzhou, China; 2 Guangdong Province Key Laboratory of Waterfowl Healthy Breeding, Guangzhou, Guangdong, China

**Keywords:** photoperiod, melatonin, reproductive function, testicular function

## Abstract

Photoperiod is an important environmental factor affecting animal physiological function. Melatonin is an endogenous hormone that plays an important role in circadian and seasonal (or cyclical) rhythms and seasonal reproduction in mammals. To investigate the effects of melatonin on the reproductive performance of adult male mice under different photoperiods, sixty mice were randomly allotted to six groups: control (Light Dark, 12 L:12 D), control plus melatonin (MLD, 12 L:12 D), 24-hour continuous light (LL, 24 L:0 D), 24-hour continuous light plus melatonin (MLL 24 L:0 D), constant darkness (DD, 0 L:24 D), and constant darkness plus melatonin (MDD, 0 L:24 D). Normal saline (100 μL) was injected into the LD, LL, and DD groups at noon each day; the MLD, MLL, and MDD groups were injected with melatonin (1 mg/mL; 2 mg/kg·body weigh). After 24 hours of prolonged light exposure, testis morphology decreased, convoluted seminiferous tubules became sparse, the diameter of convoluted seminiferous tubules decreased, and the level of sex hormones decreased. After the administration of exogenous melatonin, testicular morphology and sex hormone levels decreased in the MLD group under normal light conditions. In the MLL group, the testicular tissue morphology returned to normal, the diameter of convoluted tubules increased, the hormone levels of LH (Luteinizing hormone) and MTL (melatonin) significantly increased (*P*<0.05), and th0e gene expressions of *LHβ* and *Mtnr1A* (*Melatonin receptors 1A*) increased. There was almost no difference in the MDD group under continuous darkness. In conclusion, melatonin can damage the reproductive performance of male mice under normal light conditions, while exogenous melatonin can alleviate and protect the testicular injury of male mice under continuous light conditions.

## Introduction

Melatonin is an important, endogenous, rhythmic hormone secreted by the pineal gland, which regulates the circadian and seasonal rhythms of the body and is one of the key hormones affecting the reproductive physiology of seasonally reproducing mammals. Melatonin is produced during the dark phase of the circadian rhythm, and light signals can have an inhibitory effect on melatonin synthesis and secretion that in turn affects animal activity and reproductive physiological processes (Simonneaux et al., 2009; Abecia et al., 2017; Talpur et al., 2018; Vivid and Bentley, 2018). Photoperiod is a key environmental factor that regulates seasonal reproduction in rodents (Malpaux et al., 2001). Studies have shown that long periods of light suppress melatonin levels in young Turkish hamster pups leading to suppressed gonadal development, while melatonin and short light periods led to decreased body weight and gonadal weight and significantly suppressed testicular development in Siberian hamsters and mice (Pitrosky and Pévet, 1997; Malpaux et al., 1999; Ono et al., 2008; Russart and Nelson, 2018). These results indicate that the effects of light and melatonin on the reproductive performance of seasonal animals has ecological significance (Dominoni et al., 2013; Robert et al., 2015). For seasonal animals, light can influence the effect of melatonin on reproductive activity. Melatonin stimulates sexual activity in short-day breeding animals, while it inhibits activity and reproductive physiological processes in long-day breeding animals (Tan et al., 2014; Tian et al., 2017; Yu et al., 2018). In animal photoperiodic responses, melatonin is an important intermediate responsible for the conversion of light signals into endogenous neuroendocrine signals that regulate the activity of the hypothalamic-pituitary-gonadal axis (HPG) (Mukherjee and Haldar, 2016). Previous studies have also shown that photoperiod can regulate the secretion of testosterone and increase testicular volume by regulating the secretion of melatonin (Majrashi et al., 2017; Sogorescu et al., 2012). Thus, photoperiod may regulate animal reproductive activity by controlling the secretion of melatonin.

The regulation of the reproductive axis by melatonin is currently thought to be mediated through the MBH region of the hypothalamus and the pituitary nodules, which exert a regulatory effect on reproductive physiology in animals by binding to their specific membrane-bound receptors (MT1, MT2 and MT3) (Charif et al., 2022; Talpur et al., 2018; Tan et al., 2014; Tian et al., 2017). MT1 is mainly distributed in the suprachiasmatic nucleus of the hypothalamus. The expression of MT1 inhibits the secretion of GnRH and thus the release of luteinizing hormone (LH), which affects the reproductive function of animals. In males, melatonin acts mainly through the regulation of two key neurohormones, GnRH secreted by the hypothalamus and LH secreted by the pituitary gland (Livak and Schmittgen, 2001; Martin et al., 1980). LH acts mainly on testicular Leydig cells and regulates testicular growth and spermatogenesis (Spaliviero et al., 2004; Dubocovich and Markowska, 2005). Other studies have found that melatonin can acutely induce increased expression of *RFRP-3*, thereby regulating reproductive function (Ansel et al., 2011; Vivid and Bentley, 2018). In addition, studies have shown that melatonin can directly regulate ovarian and testicular functions and affect animal reproductive functions by activating complex mechanisms. Injection of MLT into the testicles of mice significantly reduced testosterone levels, testicular volume, and androgen synthesis (Ahmad and Haldar, 2010; Qin et al., 2015). At the gonadal level, melatonin regulates testicular growth, cAMP production, and testosterone production (Olivares et al., 1989; Touitou et al., 1992; Valenti et al., 1995). The down-regulation of melatonin receptors, especially MT1, leads to a significant decline in testosterone levels (Dubocovich and Markowska, 2005; Yu et al., 2018; Gao et al., 2019). It has also been reported that melatonin can be involved in animal reproductive regulation by regulating the expression of receptors in testes (McGuire et al., 2011).

Photoperiod induces changes in animal reproductive physiology by regulating the secretion of melatonin. In modern society, due to urbanization, many cities are never dark. Animals may have to endure the effects of artificial light for very long periods of time, resulting in insufficient secretion of endogenous melatonin, which seriously affects reproductive performance. Therefore, the purpose of this study was to analyze the effects of exogenous melatonin on testicular development and genes related to reproduction in male adult mice under different photoperiods, to understand the effects of light and melatonin on animal reproductive performance.

## Methods

### Animals and experimental design

**Figure 1:**
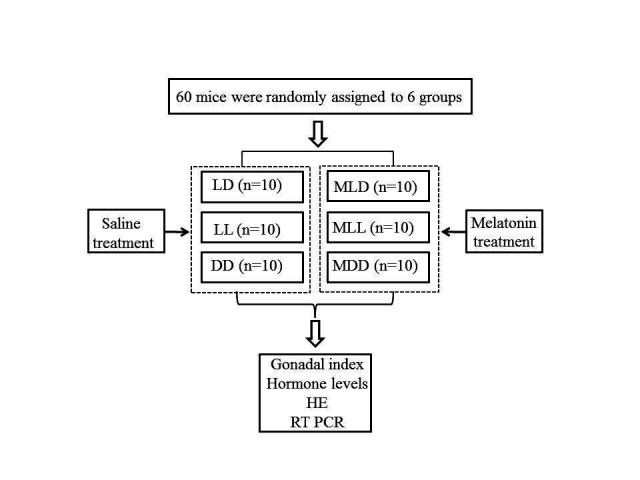
Experimental treatment of the mice.

All operations in this study followed the procedures no.2019101013 approved by the Institutional Animal Care and Use Committee of Zhongkai University of Agriculture and Engineering. We selected sixty 49-day-old healthy male mice with the same body weight (SPF grade; purchased by the Experimental Animal Center of Guangzhou University of Chinese Medicine; animal license: SCXK 2018-0034). The mice were placed in the same room, light-controlled 12L:12D, each mouse in a separate cage (Otálora et al., 2008), at 25°C±3°C, 40%–70% relative humidity, and ≤15 mg/m^3^ ammonia concentration. The bedding was changed every day, and an adequate food diet and water was provided. The mice were randomly divided into six groups (*n* = 10): control (LD, 12L:12D), control plus melatonin (MLD, 12L:12D), 24 h light (LL, 24L: 0D), 24 h light plus melatonin (MLL, 24L:0D), complete darkness (DD, 0L:24D), and complete darkness plus melatonin (MDD, 0L:24D). After seven days of adaptive feeding. The experiment started at 56 days of age and ended at 66 days of age, and the experiment lasted 10 days. According to the light program, 100 μL of normal saline was injected into LD, LL, and DD at noon every day, while MLD, MLL, and MDD were injected with 100 μL concentrationof 1 mg/mL melatonin (2 mg/kg·BW) (Otálora et al., 2008; Ahmad and Haldar, 2010; Qin et al., 2015) (Figure 1). All the experimental groups had the same feeding and management conditions. They were drinking water and taking food freely. Observations were taken of their feeding, spirit, activity, appetite, health, etc., and the number of deaths and causes were also recorded. On the last day of the experiment, blood was collected within 30min after melatonin injection (Wang et al., 2018) and the serum was shared to determine the concentrations of testosterone (T), luteinizing hormone (LH), and serum melatonin (MT). Meanwhile, hypothalamus and pituitary glands were extracted to detect the expression of related reproductive genes. All mice were treated in a humane manner.

### The testis index assay

The weights of testes were measured immediately after the mice were euthanized. The testis index is the ratio of testis weight (g) to body weight (g) multiplied by 100%.

### Histological observation and analysis of testis

The paraffin-fixed blocks were serially sectioned into 5- to 6-μm-thick coronal slices. For routine histological examination, the paraffin sections were stained with HE (hematoxylin-eosin staining). HE-stained slices were analyzed under a Nikon fluorescence microscope (Nikon, Tokyo, Japan). Testicular tissue section observation and analysis: The testicular tissue screenshots of 5.0×, 10.0×, 20.0×, and 40.0× were taken from the CaseViewer to observe the changes of seminal duct diameter, spermatogenic cells and interstitial cells. Image-pro Plus 6.0 analysis software was used to measure the diameter of five varicospermal tubules (μm) in each 5.0× screenshot, using μm as the standard unit. For quantification, three randomly selected HPFs were analyzed in each section. The number of Leydig cells (20.0× screenshots) in each section was calculated, the field area (mm^2^) was calculated, and the density of Leydig cells (mm^2^) were calculated. The number of spermatogenic cells at all levels in three 40.0× screenshots in each section were recorded. Quantitative data are presented as mean standard error. Analysis of variance was used for sufficient statistical analysis (Yao et al., 2004; Choi, 2013; Akhtar et al., 2019; Zhang et al., 2020).

### Serum hormone levels assay

LH (Luteinizing hormone), MTL (melatonin), and T (testosterone) in serum were measured by means of enzyme-linked immunosorbent assay (ELISA) kits (DECO; bought from Shanghai Huiying Biological Technology Co. Ltd). These kits were Mouse luteinizing hormone ELISA Kit (sensitivity: 25ng/L; detection range: 100-2000ng/L;), Mouse melatonin ELISA Kit (sensitivity: 5ng/L; detection range: 20-480ng/L), Mouse testosterone ELISA Kit (sensitivity: 2ng/L; detection range: 8-240ng/L). All measurement methods were carried out in strict accordance with the instructions.

### Quantitative reverse-transcription PCR analysis

Hypothalamus and pituitary samples were collected from the same location, frozen in liquid nitrogen, and stored at -80°C for total RNA collection from the hypothalamus, pituitary, and testis tissues. All tissue samples were extracted using the same RNA extraction method. The tissues were added to a 2 mL EP tube, and then 1 mL Trizol(Invitrogen, Foster City, CA, USA) was added, repeatedly clipped, and a magnetic bead was placed and the tissue was thoroughly broken using a tissue crusher. Add 200 μL of pre-cooled chloroform to each tube and let stand for 3 min at 4°C in a centrifuge, then centrifuge for 15 min at 4°C and 12000 rpm. Carefully aspirate 500 μL of the upper layer into a 1.5 mL EP tube without RNA, add an equal volume of isopropanol and let stand for 5 min on ice, then centrifuge for 10 min at 4°C and 12000 rpm to obtain the bottom of the tube. Add 1 mL of 75% ethanol (DEPC water preparation) and centrifuge at 4°C and 7500 rpm for 10 min; decant the ethanol supernatant, dry the EP tube upside down on absorbent paper for 3 min, and add 25 μL of DEPC-treated water to dissolve the RNA precipitate. Then reverse-transcribed to synthesize the first strand cDNA using a ReverTra Ace qPCR RT kit (Toyobo, Osaka, Japan). Quantitative real-time PCR (qPCR) was performed in a 20 μl reaction volume using SYBR Green PCR Master Mix (Invitrogen) and 2.5 pmol primers using an ABI 7500 system (Applied Biosystems, Foster City, CA, USA). The primers for the quantitative real-time PCR (RT-qPCR) were designed using the mRNA sequences of the related genes in mouse (Table 1) and then synthesized by Sangon Biotech (Shanghai) Co., Ltd. The relative expression levels of the gene were calculated using the comparative 2^−ΔΔCT^ (CT is threshold cycle) method as previously described (Livak and Schmittgen, 2001; Majrashi et al., 2017).

**Table 1:** Primer sequences for quantitative PCR.

**Gene name**	**Primer sequences (5’-3’)**	**Annealing temperature (°C)**	**PCR product (bp)**	**Accession** **number**
***GnRH***	F:CGGCATTCTACTGCTGACTGTG	61	215	XM_021182616.1
	R:GCCTGGCTTCCTCTTCAATCA			
***LHβ***	F:GTGAGCCCAAGTGTGGTGT	58	97	XM_011250810.2
	R:GATGCTGGTGGTGAAGGTGA			
***Mtnr1A***	F:GTGGAGAGTTCAAATGAAGAAGCA	60	159	NM_008639.2
	R:GAAAGCAAACAAGGAGAGCAGTAAG			
***β-actin***	F:AGATCAAGATCATTGCTCCTCCT	60	151	XM_021163894.1
	R:ACGCAGCTCAGTAACAGTCC			

F represents the forward primer; R represents the reverse primer.

### Statistical analysis

The differences among treatments were statistically analyzed with one-way analysis of variance (ANOVA) tests in a randomized design, using SPSS Statistics 19.0. Values were presented as the mean ± standard error of mean (SEM). The threshold for significance was set at *P* < 0.05 and for high significance at *P* < 0.01.

## Results

### Body weight and testicular index

In this study, there were no significant differences in both body weight and testicular index between groups. After the exogenous MLT injection, body weight gain in both MLD and MLL groups was lower than in the LD and LL groups, and body weight gain in the MDD group was higher than in the DD group, but the difference was not statistically significant (*P* >0.05). The trend in change in the testicular index was similar to that of body weight, and the LD group was higher than the LL and DD groups, but there were no significant differences between the three groups (Table 2).

**Table 2:** Effect of photoperiod on body weight gain and gonadal index.

	**LD**	**MLD**	**LL**	**MLL**	**DD**	**MDD**
Body weight (g)	48.35±0.85	47.78±1.58	45.80±1.88	46.05±1.09	46.25±1.49	45.65±1.14
Gonadal index (%)	0.60±0.04	0.60±0.05	0.56±0.05	0.57±0.03	0.54±0.05	0.58±0.05

### Development of seminiferous tubules

**Figure 2:**
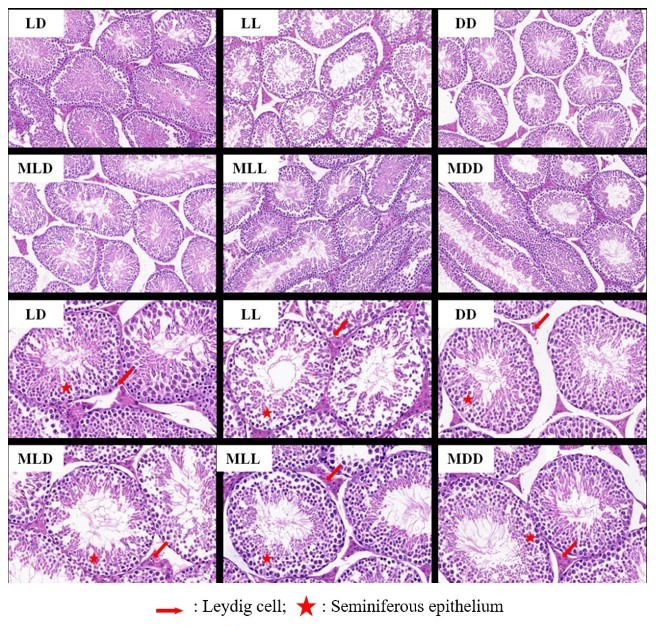
Effect of photoperiod on the development of testis (A,B:×200;C,D:×400). LD: control group; MLD: control plus melatonin group; LL: 24 h light group; MLL: 24h light plus melatonin group; DD: complete darkness group; MDD: complete darkness plus melatonin group.

The seminiferous tubules were clearly visible and spermatogenic cell layers were arranged neatly and well development in the LD group. However, in the LL group, the spermatogenic epithelial cells decreased in thickness, the number of cell layers decreased significantly, and there was almost no sperm found in the tubules. The interstitial space in the DD group was larger than that in the LD group. Following the injection in the MLT groups, there were no significant differences between the LD and MLD groups in morphological characteristics of principal, basal, and epithelial cells in the duct of the epididymis. The convoluted tubule epithelial cells in the MLL group were more multilayered and arranged regularly than those in the LL group, and there was no significant difference between the MDD group and the DD group in overall morphological structure (Figure 2).

**Figure 3:**
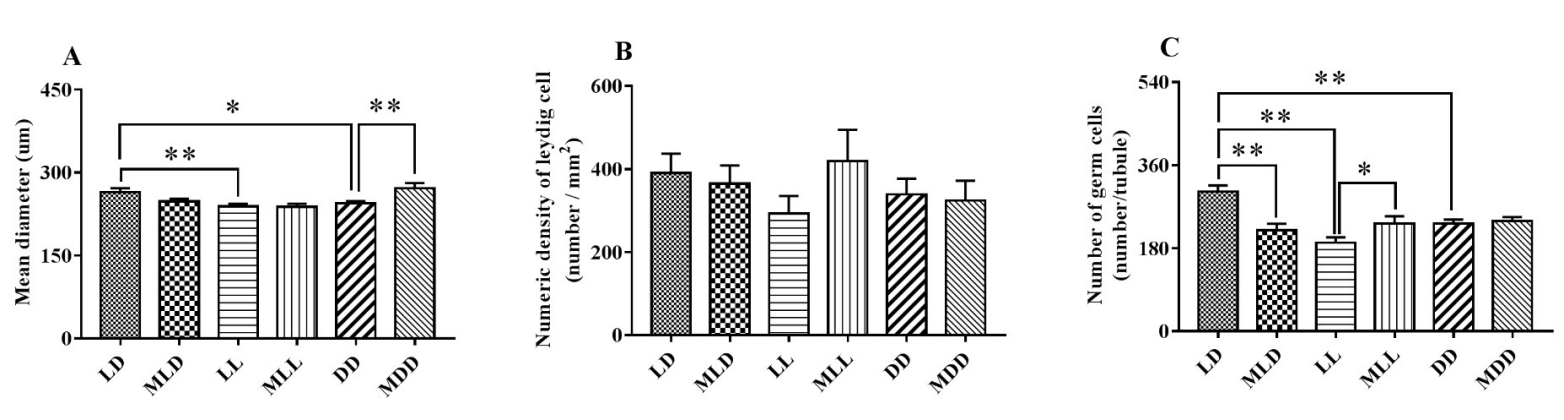
Effect of photoperiod on convoluted tubule histological index. (A) Effects of exogenous melatonin and different photoperiods on the diameter of convoluted seminiferous tubules; (B) effects of exogenous melatonin and different photoperiods on Leydig cell density of convoluted tubules; (C) effects of exogenous melatonin and different photoperiods on the number of convoluted germ cells. Data are expressed as the mean ± SEM, n = 4. *P < 0.05; **P < 0.01.

The diameter of seminiferous tubules in the LD group was significantly longer than in the LL and DD groups (*P* <0.05). Leydig cell density showed no significant differences among other treatment groups (*P* >0.05). The number of spermatogenic cells in the LD group was significantly higher than in the LL and DD groups (*P* <0.05), the number of spermatogenic cells in MLD group was significantly lower than in the LD group (*P* <0.05), and the number of spermatogenic cells in the MLL group was significantly higher than in the LL group (*P* <0.05) (Figure 3).

### Hormone levels

**Figure 4:**
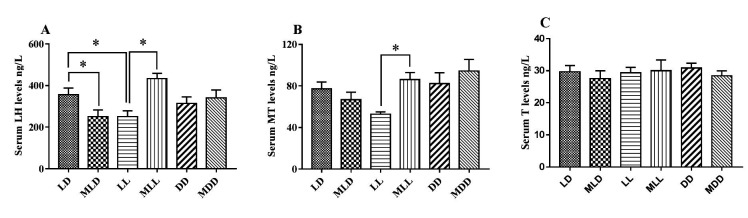
Hormone levels in male mice. (A) Serum luteinizing hormone (LH) levels; (B) serum melatonin (MT) levels; (C) serum testosterone (T) levels. Data are expressed as the mean ± SEM, n = 10. *P < 0.05; **P < 0.01.

Compared with the LD group, the LL group had significantly reduced LH (*P* <0.05) and the DD group underwent a slight decrease in LH levels (*P* >0.05). Following exogenous melatonin injection, Level of LH in MLD group found significantly lower than that in LD group (*P* <0.05). The MLL group had a significantly higher level of LH compared with the LL group (*P* <0.05). In addition, there were no statistically significant difference of the LH level between MDD group and DD group (*P* > 0.05). The MLL group had a significantly higher level of MT compared with the LL group (P <0.05). The six groups showed no significant differences in T level (*P* >0.05) (Figure 4).

### Gene expression

**Figure 5:**
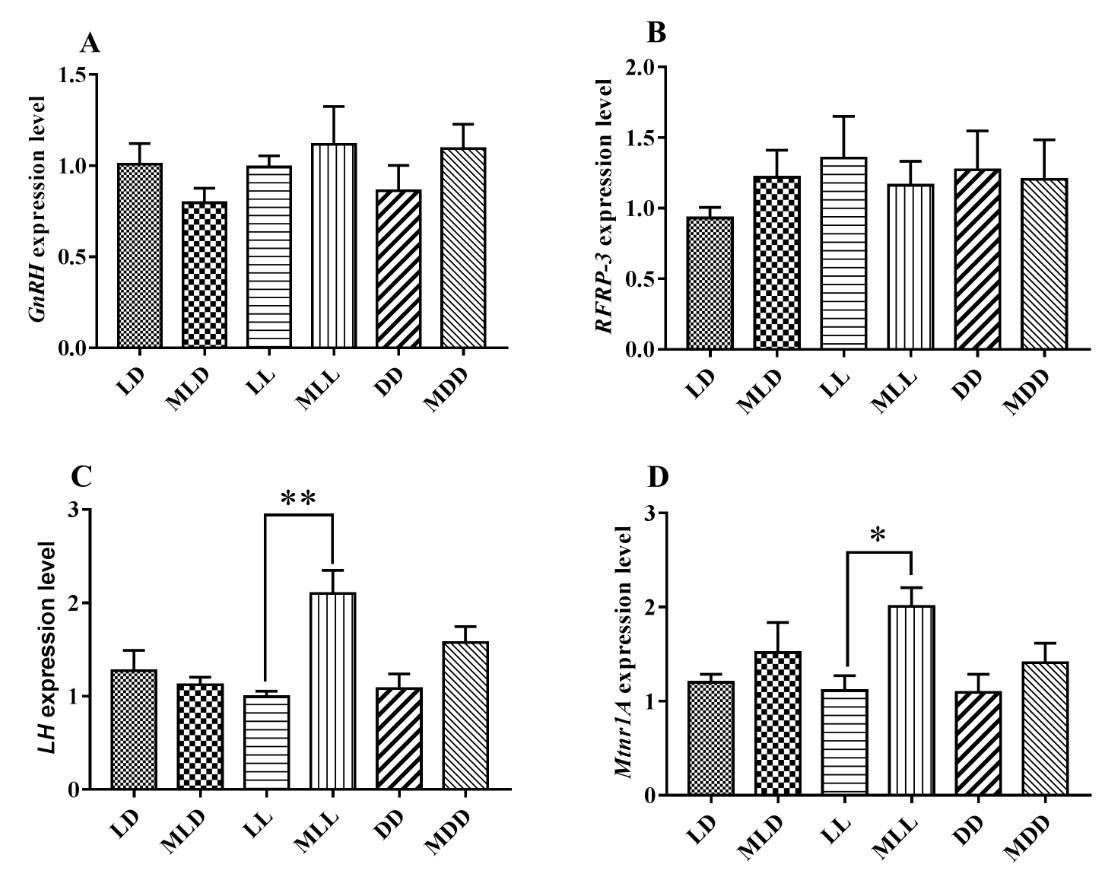
Effects of different photoperiods on the expression of reproduction-related genes. (A) Relative mRNA expression of *GnRH* in the hypothalamus; (B) relative mRNA expression of *RFRP-3* in the hypothalamus; (C) relative mRNA expression of *LH* in the pituitary; (D) relative mRNA expression of *Mtnr1A* in the pituitary. Data are expressed as the mean±SEM, n=6. * *P*<0.05; **P <0.01.

Compared with the LD group, the gene expression levels of *LHβ* and *Mtnr1A* in the LL group were decreased, while the gene expression levels of *RFRP-3* were increased, but the differences were not significant (*P* >0.05). Under normal light conditions, *GnRH* and *LHβ* gene expression decreased after MLT injection while *RFRP-3* and *Mtnr1A* gene expression increased, but the differences were not significant (*P* >0.05). The expression levels of *LHβ*, *Mtnr1A*, and *GnRH* genes in the MLL group were higher than in the LL group, and the expression levels of *LHβ* and *Mtnr1A* were significantly higher than in the LL group (*P* <0.05). Compared with the DD group, the expression of *LHβ*, *Mtnr1A,*, and *GnRH* genes increased in the MDD group, while the expression of the *RFRP-3* gene decreased, but not statistically significantly (*P* >0.05) (Figure 5).

## Discussion

Melatonin is a key hormone affecting circadian rhythms and regulating the reproductive function of animals. Darkness promotes its synthesis by activating receptor sites within the hypothalamic-pituitary-gonad (HPG) axis (Shi et al., 2013), and influences the seasonal release of GnRH through the Kisspeptin-GPR54 system (Simonneaux et al., 2009). Our results showed that 24 hours of continuous light partly reduced the male mouse gonad index, caused the seminiferous tubules to be loosely organized and their diameters to decrease, and LH and MT hormone levels decreased. Meanwhile, the expression of the melatonin receptor gene in the pituitary decreased. Thus, the effect of melatonin on reproductive physiology is not only through the inhibition of hypothalamic GnRH released and thus anterior pituitary LH secretion, but it may also act directly on reproductive organs and exert a regulatory effect on reproductive function in mammals. Frungieri et al. (2005) also found that melatonin acts on testicular interstitial cells, stimulates the expression of testicular melatonin receptors, downregulates the expression of key enzymes for steroid synthesis, inhibits androgen secretion, and reduces reproductive performance (Frungieri et al., 2005).

The addition of exogenous melatonin can significantly alleviate testicular damage caused by prolonged exposure to light. Previous studies have also shown that prolonged exposure to light causes declines in gonadal index and sperm count in mice (Sinhasane and Joshi, 1998), while melatonin can effectively improve testicular weight and testosterone levels in male mice with reproductive decline (Mirhoseini et al., 2019; Saide et al., 2019). Prolonged exposure to light causes circadian desynchrony and thereby increases the activation of the hypothalamus-pituitary-adrenal (HPA) axis which, consequently, increases the production of corticosterone. The elevated level of corticosteroids results in a reduction in testosterone production (Lateef and Akintubosun, 2020). Melatonin partly attenuates the adrenocortical response to stress, and exerts a corresponding inhibitory effect on the synthesis and secretion of the hypothalamic adrenocorticotropic hormone-releasing hormone. Therefore, melatonin plays a moderating role in the damage caused by prolonged exposure to light. In addition, melatonin is an important mediator of light-induced changes in animal performance, and there is a significant negative correlation between light exposure and melatonin concentration in vivo. Appropriate amounts of exogenous melatonin can neutralize the effects of long-term light exposure and regulate the physiological rhythms of the body. Therefore, exogenous melatonin can alleviate the damage of reproductive function caused by continuous artificial light.

However, melatonin injection under normal light conditions could lead to changes in the convoluted tubules of male mice, decreases in LH hormone levels and gene expression, and impaired reproductive ability. These results were consistent with previous findings that long-term exposure to light can promote GnIH (RFRP-3) release and inhibit GnRH release in seasonally bred animals, thus regulating reproductive function (Kelestimur et al., 2012). Supplementation of melatonin under normal light conditions can shorten light exposure and change the secretion mode of melatonin, which has a certain influence on the reproductive function of male mice.

Previous studies have shown that a sustained increase in melatonin under short light conditions would induce gonadal degeneration (Choi, 2013). However, the results of the present study show that sex hormone levels and gene expression in male mice are reduced but not significantly affected by continuous darkness, probably due to the fact that short-term darkness promotes melatonin secretion (Blank and Freeman, 1991; Miera et al., 2020), but does not have much effect on individual mice due to their own endogenous regulatory mechanisms, while exogenous melatonin injection did not have much effect on their reproductive indexes. In addition, the exogenous melatonin injection did not have a significant effect on the reproductive indexes of the mice, demonstrating that the supplementation of exogenous melatonin under dark conditions does not have much influence on reproduction.

## Conclusions

Sustained exposure to long light inhibits LH secretion and reduces the reproductive performance of mice, while exogenous melatonin alleviates the decline of LH and MT hormones under long light and upregulates *LHβ* and *Mtnr1A* genes under long light, thus alleviating the decline of reproductive function caused by long-term extreme complete long light. However, under normal light, excessive intake and administration of exogenous melatonin can reduce the inhibition of LH secretion and reduce the reproductive function of adult mice.
